# Self-Rated Health as an Independent Predictor of All-Cause Mortality in Patients With Non-ST-Segment Elevation Myocardial Infarction

**DOI:** 10.31083/RCM47517

**Published:** 2026-07-20

**Authors:** Shan Cao, Tianshu Gu, Sutao Hu, Junyu Liu, Tong Liu, Kangyin Chen

**Affiliations:** ^1^Tianjin Key Laboratory of Ionic-Molecular Function of Cardiovascular Disease, Department of Cardiology, Tianjin Institute of Cardiology, The Second Hospital of Tianjin Medical University, 300211 Tianjin, China

**Keywords:** self-rated health, non-ST-segment elevation myocardial infarction, clinical prognosis, all-cause mortality

## Abstract

**Background:**

This investigation aimed to examine the association between subjective health perception and mortality risk in individuals diagnosed with non-ST-segment elevation myocardial infarction (NSTEMI).

**Methods:**

This study conducted a multicenter retrospective analysis of 29,465 patients with NSTEMI from 82 tertiary and secondary hospitals in Tianjin, China (2010–2023). Patients were stratified into self-rated health (SRH)-satisfied (n = 28,835) and SRH-dissatisfied (n = 630) cohorts based on self-reported health assessments. Propensity score matching (1:3 ratio) was used to balance baseline characteristics, yielding a final sample of 2514 patients. The primary outcome was all-cause mortality over a median follow-up of 1477 days. Multivariable Cox regression was used to identify predictors of mortality, and Kaplan-Meier analysis was performed to compare survival between groups.

**Results:**

The SRH-dissatisfied cohort exhibited advanced age, reduced percutaneous coronary intervention (PCI) utilization, and greater comorbidity burden (diabetes, hypertension, chronic kidney disease, cerebrovascular/peripheral vascular disease, chronic lung disease; *p* < 0.05). This group demonstrated significantly elevated mortality (adjusted Hazard Ratio [aHR] = 1.29, 95% confidence interval [CI]: 1.16–1.44; *p* < 0.001). No between-group differences were observed for secondary endpoints: nonfatal myocardial infarction (MI), cardiac death, ischemic stroke, or revascularization. Subgroup analyses stratified by age, sex, and comorbidity status confirmed the consistency of the results.

**Conclusions:**

Self-rated health independently predicts mortality risk in patients with NSTEMI.

## 1. Introduction

Over recent decades, cardiovascular disorders have shown a marked rise in prevalence, with acute myocardial infarction (AMI) persisting as one of the principal contributors to worldwide mortality [1]. Meanwhile, the rising incidence of non-ST-segment elevation myocardial infarction (NSTEMI) [2,3] has made it an important public health issue worldwide. NSTEMI is a type of acute coronary syndrome, often caused by partial obstruction of the lumen, with a more widespread burden of atherosclerotic plaques in the coronary arteries [3]. This leads to its more subtle clinical presentation, often resulting in delayed diagnosis and is associated with higher long-term mortality [4]. Although significant progress has been made in the early treatment of NSTEMI, the prognosis of patients remains influenced by various factors, including multiple biomarkers and clinical characteristics [5,6,7].

Self-rated health (SRH) is a health assessment tool based on an individual’s subjective perception, typically involving a simple questionnaire in which individuals evaluate their overall health status. The concept of SRH was first introduced into health research in the mid-20th century and has since been widely applied in epidemiology, public health, and clinical research. SRH is closely related not only to an individual’s physiological health but also serves as an effective predictor of disease incidence, mortality, and other factors. It is currently widely used in the research and management of chronic diseases [8].

In the field of cardiovascular diseases (CVD), studies have confirmed that low SRH is associated with an increased incidence of heart disease [8,9] and serves as a favorable predictor for cardiovascular diseases [10]. Moreover, low SRH is linked to higher cardiovascular mortality, even in patients without cardiovascular diseases themselves [11]. Although SRH has been widely used in cardiovascular disease research, its application in NSTEMI patients remains relatively limited, and there is a lack of in-depth exploration regarding the impact of SRH on clinical outcomes in NSTEMI patients. Therefore, this study aims to evaluate the effect of SRH on all-cause mortality and other clinical outcomes in NSTEMI patients, and further analyze the relationship between the clinical characteristics and prognosis of patients dissatisfied with SRH.

## 2. Materials and Methods

### 2.1 Data Sources and Study Participants

The data for this study were sourced from the Tianjin Coronary Artery Disease (CAD) Specialized Database. The details of the platform have been described in our previous publications [12,13]. Briefly, the CAD specialized database includes patients who were hospitalized with discharge diagnoses including CAD. All healthcare information for these patients is collected, including demographic characteristics, disease diagnoses, medication and non-medication prescriptions, examination and laboratory test results, surgical information, cost details, community medication and health examination information, and public health mortality information. All exposure and outcome variables were subjected to manual sampling cross-verification between our CAD specialized database records and source medical documentation to ensure data authenticity.

**Fig. 1.**
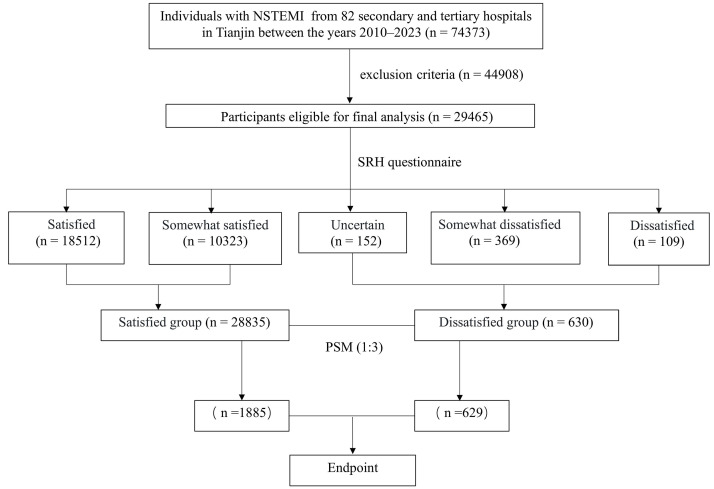
**Study flow chart**. NSTEMI, non-ST-segment elevation myocardial infarction; SRH, self-rated health; PSM, propensity score matching.

This retrospective included 29,465 patients with NSTEMI from Tianjin CAD Specialized Database between 2010 and 2023 (Fig. 1). Inclusion criteria: Patients were eligible for inclusion if they were (1) aged ≥18 years, (2) discharged with a diagnosis of NSTEMI, and (3) had complete demographic and clinical information available in the database. Exclusion criteria: Patients were excluded if they (1) had a concurrent diagnosis of malignant tumor, (2) were pregnant, (3) had missing baseline data or (4) had an absence of SRH questionnaire records in the database. This research protocol received formal ethical clearance from the Institutional Review Board at The Second Hospital of Tianjin Medical University (Ethical approval number: KY2025K010). Given the retrospective design of this analytical study, the requirement for obtaining individual patient consent was waived in accordance with established ethical guidelines for secondary data analysis.

### 2.2 Clinical Characteristics and Outcomes

Clinical characteristics were retrospectively extracted from the electronic medical records. The variables collected included demographic information, comorbidities, and in-hospital treatments. Demographic information consisted of age and sex. The study population exhibited a range of comorbidities, including hypertension, diabetes mellitus (DM), atrial fibrillation (AF), chronic kidney disease (CKD), chronic lung disease, cerebrovascular disease, peripheral vascular disease, depression, and anxiety. During hospitalization, patients received various pharmacological and interventional treatments. These encompassed antiplatelet agents, angiotensin-converting enzyme inhibitor or angiotensin II receptor blocker (ACEI/ARB), β-blocker (BB), angiotensin receptor-neprilysin inhibitor (ARNI), calcium channel blocker (CCB), statins, diuretics, nitrates, and anticoagulation. Additionally, procedural interventions such as percutaneous coronary intervention (PCI) were performed during the hospital stay.

The primary endpoint event was all-cause mortality, while the secondary endpoint events included nonfatal myocardial infarction (MI), cardiac death, ischemic stroke and revascularization.

### 2.3 Assessment of SRH

SRH was evaluated based on questionnaire data extracted from the electronic medical records prior to the occurrence of NSTEMI. The SRH questionnaire consisted of a single item asking patients to rate their overall health status on a five-point Likert scale: “satisfied”, “somewhat satisfied”, “uncertain”, “somewhat dissatisfied”, and “dissatisfied”. The number of individuals in each of the five categories was 18,512, 10,323, 152, 369, and 109, respectively. To avoid analysis bias caused by small sample sizes in the groups, we combined the last three groups into the dissatisfaction group, while the “satisfied” and “somewhat satisfied” responses were combined into a “satisfied” group. Accordingly, all patients were classified into two groups based on their SRH status: the SRH satisfied group and the SRH dissatisfied group.

### 2.4 Statistical Methods

Normally distributed continuous variables were expressed as means with standard deviations (mean ± SD), with between-group differences assessed through independent *t*-tests. For non-normally distributed continuous variables, medians and interquartile ranges (IQR) were reported, and nonparametric statistical methods were utilized for comparisons. Categorical variables were presented as proportions (%) and analyzed using chi-square tests. To minimize potential confounding effects, propensity score matching (PSM) was implemented at a 1:3 ratio between comparison groups. First, propensity scores for each patient were calculated using a logistic regression model, with the group assignment as the dependent variable. Then, the nearest neighbor matching method (caliper value = 0.01) was applied to match each patient in the ‘SRH Dissatisfied’ group with the patient in the ‘SRH Satisfied’ group who had the closest propensity score. After matching, an absolute standardized mean difference (SMD) of less than 0.1 for all variables indicated good balance between the groups. All subsequent statistical analyses were based on the matched dataset. A multivariate Cox proportional hazards regression model was used to assess the relationship between SRH and clinical outcomes. The strength of the association is expressed as a hazard ratio (HR) with a 95% confidence interval (CI). Subgroup analyses were conducted based on patients’ clinical characteristics (e.g., age, sex, comorbidities). The Kaplan-Meier estimator was employed to assess survival outcomes, with statistical significance defined as a *p*-value threshold below 0.05. Data analysis was performed using the R software, version 4.5.1 (R Foundation for Statistical Computing, Vienna, Austria).

## 3. Results

### 3.1 Baseline Characteristics of Study Participants

A total of 29,465 patients with NSTEMI were initially included, comprising 28,835 in the SRH satisfied group and 630 in the SRH dissatisfied group. Baseline characteristics presented in Table 1 revealed significant disparities prior to propensity matching. Notably, the SRH-dissatisfied cohort demonstrated advanced age, reduced PCI utilization rates, and elevated comorbidity burdens including DM, hypertension, CKD, cerebrovascular disorders, peripheral vascular disease, chronic pulmonary conditions, and psychiatric comorbidities (depression and anxiety). However, no statistically meaningful variations were detected regarding gender distribution or AF prevalence between the groups.

**Table 1.** **Basic information of NSTEMI patients**.

Characteristics		Satisfied group (n = 28,835)	Dissatisfied group (n = 630)	*p* value	Satisfied group (n = 1885)	Dissatisfied group (n = 629)	SMD
Age (y)		72.17 ± 8.12	73.97 ± 8.59	<0.001	74.33 ± 8.30	73.96 ± 8.59	0.044
Female (n, %)		12,535 (43.47)	297 (47.14)	0.072	898 (47.64)	296 (47.06)	–0.011
Comorbidities							
	DM (n, %)	11,506 (39.90)	317 (50.32)	<0.001	898 (47.64)	316 (50.23)	0.052
	Hypertension (n, %)	17,755 (61.57)	422 (66.98)	0.006	1196 (63.45)	422 (67.09)	0.076
	AF (n, %)	2302 (7.98)	60 (9.52)	0.182	171 (9.07)	59 (9.38)	0.011
	Cerebrovascular disease (n, %)	12,302 (42.66)	348 (55.24)	<0.001	1005 (53.31)	347 (55.17)	0.037
	CKD (n, %)	9549 (33.12)	248 (39.36)	0.001	720 (38.19)	247 (39.27)	0.022
	Peripheral vascular disease (n, %)	5507 (19.09)	145 (23.01)	0.016	402 (21.32)	145 (23.05)	0.041
	Chronic lung disease (n, %)	3313 (11.49)	93 (14.76)	0.013	248 (13.16)	93 (14.78)	0.047
	Depression (n, %)	702 (2.43)	26 (4.13)	0.013	49 (2.60)	18 (2.86)	0.095
	Anxiety (n, %)	1716 (5.95)	53 (8.41)	0.011	115 (6.10)	43 (6.83)	0.095
In-hospital treatment							
	PCI during hospitalization (n, %)	9725 (33.73)	114 (18.09)	<0.001	331 (17.56)	114 (18.12)	0.014
	Antiplatelets (n, %)	24,581 (85.25)	519 (82.38)	0.052	1534 (81.38)	519 (82.67)	0.033
	ACEI/ARB (n, %)	16,422 (56.95)	332 (52.69)	0.036	953 (50.56)	332 (52.78)	0.044
	ARNI (n, %)	2967 (10.29)	57 (9.04)	0.342	163 (8.65)	57 (9.06)	0.014
	BB (n, %)	16,635 (57.69)	345 (54.76)	0.153	1003 (53.21)	344 (54.69)	0.029
	CCB (n, %)	6914 (23.98)	160 (25.39)	0.437	478 (25.36)	160 (25.44)	0.002
	Statins (n, %)	23,773 (82.44)	506 (80.32)	0.182	1478 (78.41)	505 (80.29)	0.046
	Diuretics (n, %)	11,807 (40.94)	322 (51.11)	<0.001	943 (50.03)	321 (51.03)	0.020
	Nitrates (n, %)	16,909 (58.64)	371 (58.88)	0.932	1106 (58.67)	371 (58.98)	0.006
	Anticoagulation (n, %)	1081 (3.75)	22 (3.49)	0.818	53 (2.81)	22 (3.49)	0.039

PCI, percutaneous coronary intervention; SMD, standardized mean difference; AF, atrial fibrillation; DM, diabetes mellitus; CKD, chronic kidney disease; ACEI/ARB, angiotensin-converting enzyme inhibitor/angiotensin II receptor blocker; ARNI, angiotensin receptor-neprilysin inhibitors; BB, β-Blocker; CCB, calcium channel blocker.

To address potential confounding factors, we performed PSM with a 1:3 ratio comparing individuals reporting SRH dissatisfaction with those reporting satisfaction. Post-matching analysis revealed well-balanced baseline characteristics between groups, with all SMDs falling below the 0.1 threshold, indicating successful covariate balance. The pre- and post-matching baseline characteristics are presented in Table 1, with corresponding propensity score distributions visualized in **Supplementary Fig. 1**.

### 3.2 Clinical Outcomes Comparison

**Fig. 2.**
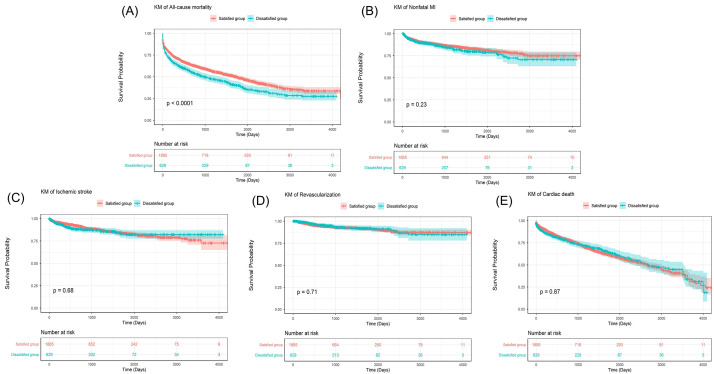
**Kaplan-Meier survival curves for outcomes between SRH satisfied and SRH dissatisfied groups**. (A) KM curve of all-cause mortality. (B) KM curve of nonfatal MI. (C) KM curve of ischemic stroke. (D) KM curve of revascularization. (E) KM curve of cardiac death. This figure illustrates the Kaplan-Meier survival curves comparing cumulative outcomes between patients with non-ST-segment elevation myocardial infarction (NSTEMI) who reported satisfied self-rated health (SRH) and those who reported dissatisfied SRH after propensity score matching (PSM, 1:3 ratio). During the median follow-up duration of 1477 days, participants reporting dissatisfaction with self-rated health status exhibited a markedly elevated mortality risk (log-rank test, *p* < 0.001). The shaded areas around the curves indicate the 95% confidence intervals. Statistical test: Kaplan-Meier survival analysis with log-rank test.

The median follow-up time was 1477 days. In the SRH satisfied and dissatisfied groups, 10,627 cases (36.85%) and 366 cases (58.09%) experienced all-cause mortality, 7397 cases (25.65%) and 184 cases (29.21%) experienced cardiac death, 3084 cases (10.69%) and 78 cases (12.38%) experienced nonfatal MI, 2751 cases (9.54%) and 61 cases (9.68%) experienced ischemic stroke, and 2053 cases (7.12%) and 30 cases (4.76%) underwent revascularization. After PSM (Table 2), the SRH dissatisfied group still showed a higher rate of all-cause mortality (58.03% vs. 47.11%, *p* < 0.001). However, there were no statistically significant differences between the groups in terms of nonfatal MI, cardiac death, ischemic stroke, or revascularization outcomes (*p* > 0.05). The Kaplan-Meier (KM) survival curves for the two groups are shown in Fig. 2.

**Table 2.** **Outcomes of different groups after PSM**.

Outcomes	Satisfied group (n = 1885)	Dissatisfied group (n = 629)	*p* value
All-cause mortality (n, %)	888 (47.11)	365 (58.03)	<0.001
Cardiac death (n, %)	554 (29.39)	183 (29.09)	0.928
Nonfatal MI (n, %)	212 (11.25)	78 (12.40)	0.476
Ischemic stroke (n, %)	183 (9.71)	61 (9.69)	1.000
Revascularization (n, %)	102 (5.41)	30 (4.77)	0.602

MI, myocardial infarction.

### 3.3 Cox Regression Analysis for All-Cause Mortality

Cox regression analysis was used to examine the relationship between SRH and clinical outcomes after PSM. Univariate analysis (Table 3) revealed that the risk of all-cause mortality (HR = 1.29, 95% CI: 1.16–1.44; *p* < 0.001) in the SRH dissatisfied group of NSTEMI patients was higher than in the SRH satisfied group. Table 4 presents the results of the univariate and multivariate Cox proportional hazards regression analyses for all-cause mortality. Based on multivariate Cox regression analysis, age is positively associated with all-cause mortality. For each additional year, the risk of death increases by 4% (adjusted hazard ratio [aHR] = 1.04, 95% CI: 1.03–1.05; *p* < 0.001). Among various comorbidities, DM and CKD are also significantly associated with all-cause mortality. Specifically, DM patients have a 19% higher risk of death compared to non-diabetic patients (aHR = 1.19, 95% CI: 1.05–1.35; *p* = 0.006), while patients with CKD have a 25% higher risk of death compared to those without CKD (aHR = 1.25, 95% CI: 1.10–1.42; *p* < 0.001). Among the various treatments included in the analysis, receiving PCI during hospitalization was independently associated with a significantly reduced risk of all-cause mortality (aHR = 0.24, 95% CI: 0.19–0.32; *p* < 0.001). Factors with *p* < 0.05 from univariate analysis were stepwise incorporated into multivariable Cox regression models for all-cause mortality, creating three models: model1, model2, and model3. Model1 only adjusted for age and sex. Model2 added the seven comorbidities (DM, Hypertension, AF, Peripheral vascular disease, Cerebrovascular disease, CKD, Chronic lung disease listed in Table 1) to model1. Model3 added the in-hospital treatment (PCI during hospitalization, Antiplatelets, ACEI/ARB, ARNI, BB, CCB, Statins, Diuretics, Nitrates) to model2. In all three models, SRH dissatisfaction remained an independent predictor of all-cause mortality (HR >1, *p* < 0.05), as shown in Table 5

**Table 3.** **Univariate Cox analysis of SRH and outcomes after PSM**.

Outcomes	Hazard ratio (95% CI)	*p* value
All-cause mortality	1.29 (1.16–1.44)	<0.001
Cardiac death	1.01 (0.86–1.20)	0.862
Nonfatal MI	1.17 (0.91–1.52)	0.225
Ischemic stroke	1.06 (0.79–1.42)	0.682
Revascularization	0.92 (0.62–1.39)	0.707

CI, confidence interval.

**Table 4.** **Cox analysis about all-cause mortality after PSM**.

Variable	Univariate		Multivariate	
	Hazard ratio (95% CI)	*p* value	Hazard ratio (95% CI)	*p* value
SRH	1.29 (1.14–1.44)	<0.001	1.33 (1.18–1.50)	<0.001
Age	1.06 (1.06–1.07)	<0.001	1.04 (1.03–1.05)	<0.001
Sex (female)	1.06 (1.00–1.11)	0.275		
Hypertension	1.32 (1.18–1.50)	<0.001		
DM	1.21 (1.08–1.35)	<0.001	1.19 (1.05–1.35)	0.006
CKD	1.67 (1.49–1.87)	<0.001	1.25 (1.10–1.42)	<0.001
AF	1.54 (1.29–1.83)	<0.001		
Cerebrovascular disease	1.37 (1.22–1.53)	<0.001		
Chronic lung disease	1.33 (1.13–1.56)	<0.001		
Peripheral vascular disease	1.26 (1.10–1.44)	<0.001		
Depression	1.03 (0.58–1.21)	0.347		
Anxiety	1.08 (0.86–1.35)	0.496		
PCI during hospitalization	0.16 (0.12–0.21)	<0.001	0.24 (0.19–0.32)	<0.001
Antiplatelet	0.46 (0.41–0.53)	<0.001	0.76 (0.63–0.94)	0.009
ARNI	0.63 (0.48–0.82)	<0.001	0.74 (0.56–0.98)	0.038
ACEI/ARB	0.55 (0.50–0.62)	<0.001	0.79 (0.69–0.91)	<0.001
BB	0.58 (0.52–0.68)	<0.001		
CCB	0.83 (0.73–0.95)	<0.001		
Statins	0.45 (0.40–0.51)	<0.001	0.70 (0.58–0.85)	<0.001
Diuretics	1.58 (1.41–1.76)	<0.001	1.70 (1.49–1.94)	<0.001
Nitrates	0.71 (0.63–0.79)	<0.001	0.81 (0.73–0.92)	<0.001
Anticoagulation	0.94 (0.67–1.34)	0.747		

**Table 5.** **Multiple Cox analysis of SRH and all-cause mortality after PSM**.

	Hazard ratio (95% CI)	*p* value
Model1	1.31 (1.16–1.48)	<0.001
Model2	1.29 (1.15–1.47)	<0.001
Model3	1.33 (1.18–1.50)	<0.001

Model1: Adjusted for age and sex; Model2: Adjusted for concomitant diseases based on model1; Model3: Adjusted for in-hospital treatment based on model2.

### 3.4 Subgroup Analysis

**Fig. 3.**
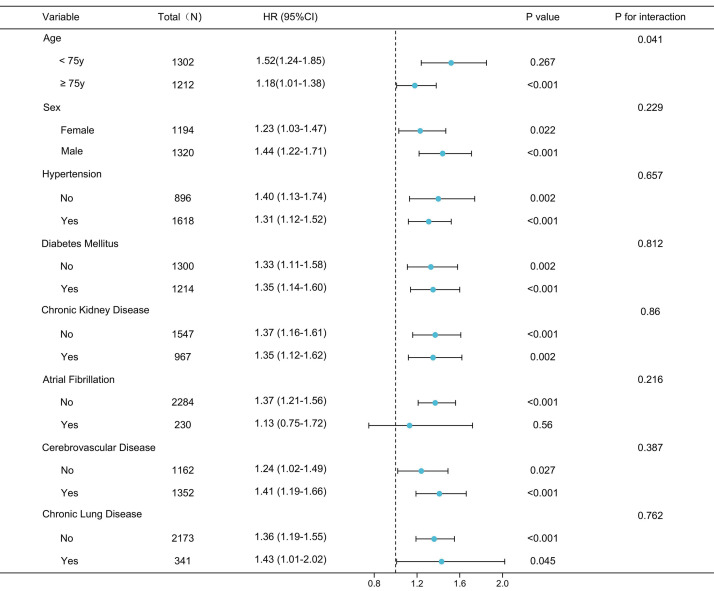
**Subgroup analyses of the association between SRH and all-cause mortality**. This forest plot presents the hazard ratios (HRs) and 95% confidence intervals for all-cause mortality comparing SRH dissatisfied versus SRH satisfied groups across prespecified subgroups, including sex, age (<75 or ≥75 years), and comorbidities (hypertension, DM, CKD, AF, cerebrovascular disease and chronic lung disease). Subgroup-specific estimates were derived from multivariable Cox proportional hazards regression models adjusted for age, sex, PCI, comorbidities, and in-hospital treatments. Statistical test: Multivariable Cox proportional hazards regression analysis. Symbols: Squares represent subgroup-specific hazard ratios; horizontal lines indicate 95% CIs; the dashed line denotes the overall HR. Abbreviations: HR, hazard ratio.

In the Cox analysis of all outcomes, all-cause mortality is the only event influenced by SRH, which is why we conducted subgroup analysis specifically for all-cause mortality. Subgroup analyses were performed according to age, sex, and major comorbidities, including hypertension, DM, CKD, AF, cerebrovascular disease, and chronic lung disease (Fig. 3). A robust positive correlation was observed between self-rated health dissatisfaction and elevated mortality risk, with this association demonstrating remarkable consistency across all pre-specified subgroup analyses. Notably, the effect appeared stronger among patients aged ≥75 years, with a significant interaction observed between SRH status and age (*p* for interaction = 0.041). No significant interactions were found for sex or other comorbidities (all *p* for interaction > 0.05).

## 4. Discussion

This retrospective cohort study, utilizing extensive clinical data collected over a 13-year period from 82 medical centers in Tianjin, demonstrated a robust correlation between SRH status and long-term mortality outcomes in NSTEMI patients. The results provide compelling evidence that suboptimal SRH serves as an independent predictor of all-cause mortality in this patient population.

SRH, defined as an individual’s subjective evaluation of their overall health status, has been extensively validated by numerous studies as a significant predictor for the incidence of various chronic diseases, including coronary heart disease, stroke, and diabetes [8]. Furthermore, SRH is significantly associated with mortality from a range of conditions [14,15,16]. Within the field of cardiovascular disease, the predictive value of SRH has also garnered considerable attention. Poor SRH not only significantly increases the incidence of cardiovascular diseases but also serves as an independent predictor of cardiovascular disease mortality [14,17,18,19,20]. A 2013 study from the UK Norfolk cohort indicated that SRH is a strong predictor of both fatal and non-fatal cardiovascular events in healthy middle-aged populations [10]. A 2014 meta-analysis incorporating 20 relevant studies found that poor SRH was associated with higher cardiovascular mortality, regardless of whether the study participants had pre-existing cardiovascular disease [20]. This conclusion is supported by large-scale prospective cohort studies. For instance, a longitudinal study in China, following 512,713 adults across 10 regions for 9.9 years, confirmed that participants with poorer SRH had significantly higher risks of all-cause mortality and cardiovascular mortality [19]. Given that cardiovascular disease represents a broad category, investigating the impact of SRH on specific cardiovascular disease subtypes could yield more targeted insights for clinical practice. Analyzing data from the China Kadoorie Biobank involving 486,541 individuals, Dong et al. [11] found that poor SRH was associated with an increased risk of incident ischemic heart disease (HR = 1.76). Similarly, a 2016 study of patients with stable heart failure found that poorer SRH was independently associated with increased mortality (HR = 1.42; *p* < 0.001) [18]. Against this backdrop, the present study focuses specifically on patients with NSTEMI. Our univariate Cox regression analysis initially indicated a significant association between SRH and all-cause mortality in this NSTEMI population. More importantly, even after comprehensive adjustment for confounders including age, sex, comorbidities, and in-hospital treatment, SRH remained a robust predictor of all-cause mortality. This finding is highly consistent with the established research narrative. Although many variables such as age, DM, CKD, or PCI during revascularization influence overall mortality, SRH is also a robust and previously undescribed predictor of all-cause mortality in NSTEMI patients. To assess the robustness of this association, we performed subgroup analyses. The results demonstrated that the predictive effect of SRH on all-cause mortality persisted across most of the eight pre-specified subgroups including those stratified by sex and by the presence or absence of hypertension, DM, CKD, or cerebrovascular disease thereby strongly reinforcing the reliability of our primary result. It is noteworthy that the association did not reach statistical significance in certain subgroups, specifically patients aged <75 years and those with comorbid AF. We posit that this may be attributable to the relatively limited sample size within these specific patient subsets.

The association between SRH and the risk of disease incidence, mortality, and other adverse outcomes has been explained in existing studies primarily through the following aspects: (1) Lifestyle Factors: Adopting beneficial lifestyle habits, including engaging in consistent exercise, consuming a nutritionally adequate diet, and abstaining from tobacco use and heavy alcohol intake, plays a crucial role in controlling metabolic and vascular risk factors including hyperglycemia, abnormal lipid profiles, and elevated blood pressure. This comprehensive approach contributes significantly to lowering the incidence of adverse cardiovascular outcomes. This is consistent with the lower comorbidity rates observed in the SRH satisfied group in this study, suggesting that a healthy lifestyle may contribute to reducing the risk of comorbidities, which in turn enhances individuals’ satisfaction with their health status. (2) Psychological State and Stress Response: Research indicates that psychological states such as depression and anxiety are important determinants of self-rated health [21]. Patients with poorer SRH typically exhibit higher levels of psychological stress, depression, and anxiety, a finding that was also confirmed in this study. These psychological states may exacerbate the burden on the cardiovascular system by activating the sympathetic nervous system and promoting the release of inflammatory factors [22], thus increasing the risk of mortality. (3) Biological Mechanisms: SRH may reflect an individual’s underlying chronic inflammatory status, immune function, and metabolic health, a theory supported by a substantial body of research. Multiple studies have found that populations with poorer SRH tend to exhibit elevated systemic inflammation levels, including higher concentrations of C-reactive protein (CRP) [23,24,25], fibrinogen [23,25], and interleukin-6 (IL-6) [24]. Furthermore, among cardiovascular risk-related proteins, inflammatory markers such as leptin and C-C motif chemokine ligand 20 (CCL20) have also been linked to poor SRH [26]. In addition to the mechanisms described above, various demographic and socioeconomic factors may influence SRH and contribute to mortality disparities. Variables such as race, ethnicity, income, and education have been shown to be significantly correlated with subjective health ratings and may act as confounders in the relationship between SRH and mortality [16].

### Limitations

While this investigation demonstrates a meaningful correlation between SRH and clinical outcomes in individuals with NSTEMI, several limitations should be acknowledged. First, as a retrospective study, the data were derived from historical medical records, which may be subject to information bias. Importantly, there were significant differences in major baseline characteristics between the SRH groups. Although we employed PSM to minimize the impact of these confounding factors, residual bias may persist due to unmeasured variables. Second, some laboratory indicators (such as cardiac and inflammatory markers) were not included due to missing data, limiting further exploration of the biological mechanisms underlying the association between SRH and all-cause mortality. Third, SRH assessment relied primarily on patients’ subjective reports without a standardized approach, potentially introducing influence from unmeasured factors such as cultural background, economic status, income level, and current mood. Additionally, merging the five-level SRH scale into two categories for analytical purposes may have introduced classification bias. Fourth, although the median follow-up period was relatively long, facilitating the observation of long-term outcomes, it also increased the risk of attrition, potentially affecting the reliability. Finally, the study population was drawn from a single hospital system in one city, with relative homogeneity in race and genetic background, which may limit the generalizability of our findings to broader populations.

## 5. Conclusions

This study shows that poor SRH is an independent predictor of all-cause mortality in NSTEMI patients, highlighting its important prognostic value. Although these conclusions need to be further validated in prospective designs and multi-center studies involving diverse racial groups, the results undoubtedly emphasize the importance of incorporating SRH into the health management and risk assessment systems for NSTEMI patients. This provides a new perspective beyond traditional biomedical indicators for clinical practice. Future research should focus on exploring effective intervention strategies based on SRH, to develop personalized management approaches and ultimately improve the long-term survival and quality of life of NSTEMI patients.

## Availability of Data and Materials

Please contact the corresponding author for access to the data.
