# Supplementary material for: Self-Rated Health as an Independent Predictor of All-Cause Mortality in Patients With Non-ST-Segment Elevation Myocardial Infarction
Source: Rev Cardiovasc Med. 2026 Jul 20;27(7):47517. doi: 10.31083/RCM47517 (PMC13419956; doi:10.31083/RCM47517)
Supplement: Supplementary file 1 [file 2153-8174-27-7-47517-s1.zip › Supplementary Material.docx]

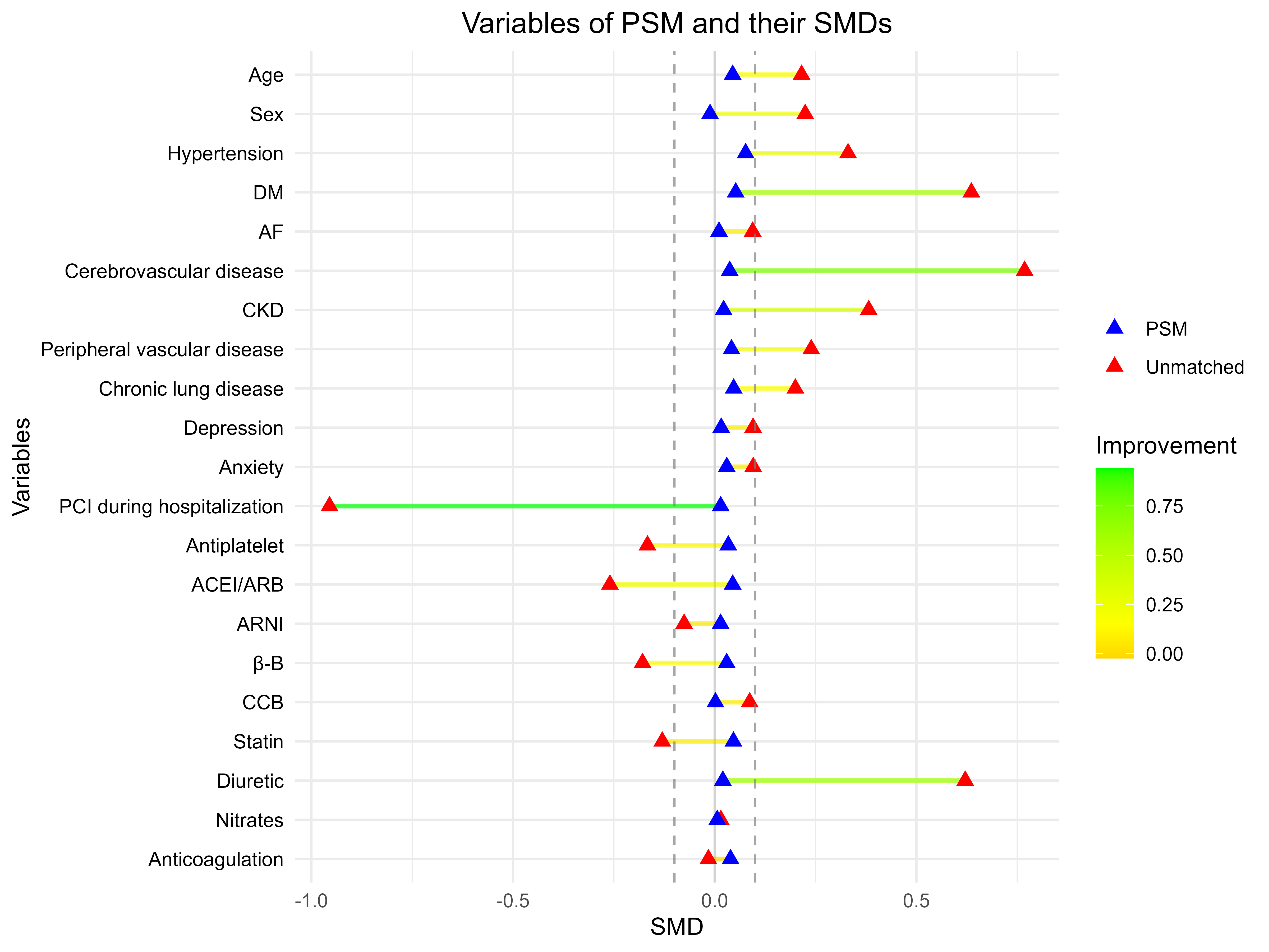
 **Supplementary Fig. 1. Variables of PSM and their SMDs**

PCI: Percutaneous Coronary Intervention; SMD: Standardized Mean Difference; AF: Atrial Fibrillation; DM: Diabetes Mellitus; CKD: Chronic Kidney Disease; ACEI/ARB: angiotensin-converting enzyme inhibitor/angiotensin II receptor blocker; ARNI: angiotensin receptor-neprilysin inhibitors; BB: β-Blocker; CCB: calcium channel blocker
